# The Effect of a Textured Insole on Symmetry of Turning

**DOI:** 10.1155/2018/6134529

**Published:** 2018-03-20

**Authors:** Etem Curuk, Yunju Lee, Alexander S. Aruin

**Affiliations:** Department of Physical Therapy, University of Illinois at Chicago, Chicago, IL, USA

## Abstract

Turning while walking is a common daily activity. Individuals with unilateral impairment frequently perform turns asymmetrically. The purpose of the study was to investigate the effect of a discomfort-inducing textured insole on symmetry of turning. Nine healthy individuals performed turns to the right while walking with no insole, immediately after the insole was inserted in the right shoe, and after walking for six minutes with the insole. The duration of turning, displacements of pelvic markers, and perceived level of discomfort were evaluated. Utilizing the insole was associated with the increased level of perceived discomfort (*p* < 0.05). Moreover, using the insole was linked to changes in the displacement of two pelvic markers and larger asymmetry index while turning immediately after the insole was inserted in the right shoe as compared to no insole condition (*p* < 0.05). The duration of right turning increased immediately after the insole was inserted (*p* < 0.05) and after walking with the insole for six minutes. The results indicate that the textured insole creates asymmetry of turning in healthy individuals. The outcome provides a background for future studies focused on using a textured insole to minimize the asymmetry of turning commonly seen in individuals with unilateral impairment.

## 1. Introduction

Thirty-five to 45% of all steps during daily life activities are made during turning movements and up to 50% of steps accounted for turning indoors [1]. While healthy persons easily perform turns in most of the cases, individuals with unilateral impairment, for example, due to a stroke, frequently experience difficulties implementing turns in their daily activity most likely due to gait asymmetry [2]. Since the turning maneuver imposes step asymmetries on an already asymmetric walking pattern reported in such individuals, people with stroke are more prone to falls and associated injuries [3, 4].

Previous studies examining the mechanisms underlying falls during turning in individuals with stroke demonstrated that these individuals exhibit delayed initiation of turns and longer time to turn and use more steps to perform the task [5, 6]. Given that there is a consensus in the literature that the ability to safely turn during walking is a key factor for safe and functional ambulation [7], it is important to design novel approaches for gait rehabilitation focusing on improvement symmetry of ambulation and turning. Several such approaches were recently introduced. Among them is the Compelled Body Weight Shift (CBWS) therapy that is based on a forced shift of body weight towards a person's affected side by means of a shoe insert that establishes a lift of the unaffected lower extremity [8, 9]. Another approach that is used to minimize asymmetry of gait is based on utilizing discomfort created by a single textured insole [10, 11]. Thus, it was shown that a discomfort-inducing insole created asymmetry of gait in healthy subjects [10, 12] and that when individuals with stroke were provided with a textured insole in the shoe on the unaffected side, the level of their asymmetry of stance and gait was reduced [10, 13].

However, no studies were conducted to evaluate the effect of a discomfort-inducing insole on symmetry of turning. Thus, the aim of the study was to investigate whether a single textured insole could alter the performance of the turning. We hypothesized that discomfort created by a textured insole will cause asymmetry of turning in healthy individuals. We also hypothesized that this asymmetry would be present after a short-term use of the textured insole.

## 2. Methods

### 2.1. Participants

Nine volunteers (age: 25.11 ± 3.17 years, 2 males, 7 females) without any neurological and musculoskeletal disorders participated in the study. All participants were right-side dominant. The experimental protocol and ethical consent forms were approved by the Institutional Review Board (IRB) of the University of Illinois at Chicago. All methods were performed in accordance with the relevant guidelines and regulations. Informed consent was obtained from all participants.

### 2.2. Instrumentation and Procedure

**Figure 1:**
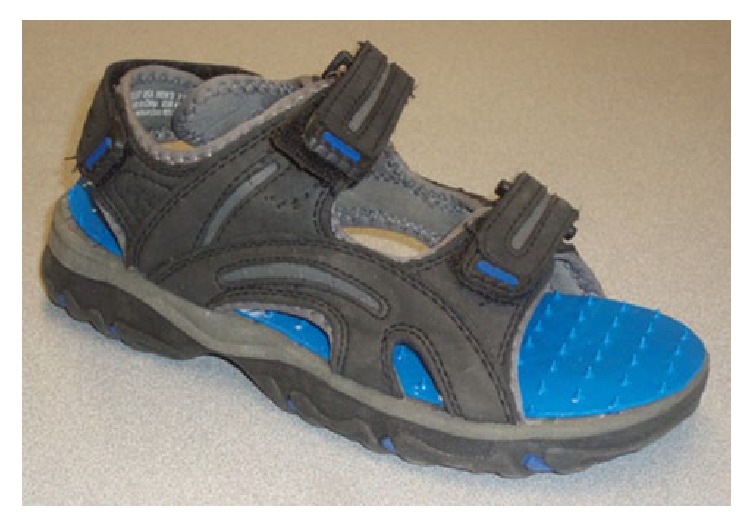
Textured insole inserted in the sandal.

The subjects were required to wear standardized sandals with no insole and with a textured insole placed in the right sandal and perform turning while walking. Standard sandals were used based on foot size; sandals also had Velcro straps allowing adjustment. During the experiments, the subjects wore standardized socks (thickness 0.6 mm, MUJI, USA). The textured insole was made of polyurethane base 1 mm high (embedded with 3 mm height and 4.3 mm base pyramidal peaks with center to center distance of approximately 16 mm) (Figure 1). The total height of the insole was 4 mm.

**Figure 2:**
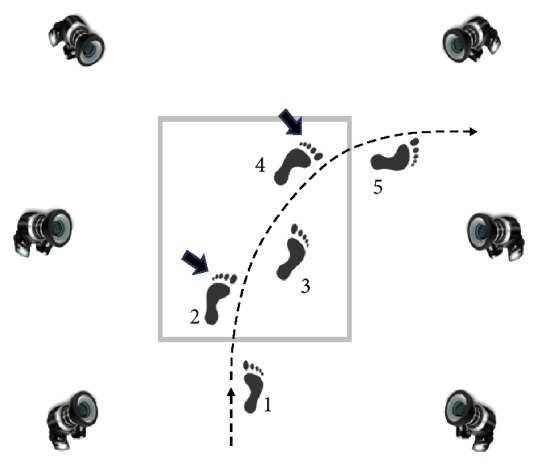
Schematic representation of the experimental set-up. The square denotes the area where the individuals took turn; turning part of the task is shown with two arrows indicating the moment of the toe-off during the second step and the toe-off during the fourth step. Step numbers are shown.

Subjects were instructed to walk along paths delineated by lines marked on the floor of the laboratory and turn 90 degrees to the right when reaching the marked turning square (Figure 2). The selection of 90 degrees' turn was based on two factors: (1) the majority of turns performed in daily life involve rotation of the body between 76 degrees and 120 degrees [14]; (2) previous research frequently involved investigation of 90 degrees' turns [15].

The assessments of turning were performed at three time points. Time point 1 (TP1) was when the subjects walked with no insole. Time Point 2 (TP2) was commenced immediately after the subjects were provided with the insole and given a one-minute period of familiarization, and Time Point 3 (TP3) assessment was commenced after 6 min of walking with the insole. In each experimental condition subjects started walking with their comfortable speed following an instruction “walk as comfortable as possible, as you walk in daily life.” To reach their comfortable speed subjects started walking 2 meters before and continued walking 2 meters after the turning event. Five turns were performed in each experimental condition. In each trial the experimenter provided the following commands: “ready,” “go,” and “relax.”

The perceived level of discomfort associated with using a textured insole was evaluated with a Visual Analog Scale (VAS) [16]. The subjects were asked to describe their level of discomfort by marking a 10 cm linear scale (with one end (0 cm) marked as “no discomfort at all” and the other end (10 cm) as “worst discomfort ever”). VAS was used in the past to assess the perceived level of comfort associated with wearing insoles [17]. High reliability has been reported for the VAS when compared with other scales used for self-assessment of comfort level [18].

Three-dimensional kinematic data was collected using a six-camera VICON 612 system (Oxford Metrics, UK). Retroreflective markers were placed over anatomical landmarks bilaterally according to the Plug-In-Gait (PIG) model (Oxford Metrics), which includes the following: second metatarsal head, calcaneus, lateral malleolus, lateral epicondyle of the femur, a marker on the lateral border of the leg (between the lateral malleolus and femoral epicondyle markers), anterior/posterior superior iliac spines, a marker on the lateral border of the thigh (between the femoral epicondyle and anterior superior iliac spines), second metacarpal, lateral epicondyle of the humerus, acromioclavicular joint, and a marker on the lateral border of the arm (between the humeral epicondyle and the acromioclavicular joint markers). Also, subjects wore head and wrists bands with four and two markers attached on them, respectively. Finally, five additional markers were attached over the following landmarks: 7th cervical vertebra, 10th thoracic vertebra, inferior angle of the right scapula, between the two sternoclavicular joints, and xiphoid process of the sternum bone. Kinematic data was acquired at 100 Hz by means of the VICON 612 data station.

### 2.3. Data Analysis

Trajectories of markers were derived from the VICON system. Initial processing of the kinematic data was done using the VICON software to detect the start and end points of each turning trial. MATLAB software (MathWorks, USA) was used for offline data processing. Kinematic analysis was focused on the analysis of the displacement of pelvic markers. It was reported in the literature that positions of the pelvic markers could represent the displacements of the center of mass (COM) of the body [19]. Thus, data obtained from the four pelvic markers, Left Anterior Superior Iliacus (LASI), Right Anterior Superior Iliacus (RASI), Left Posterior Superior Iliacus (LPSI), and Right Posterior Superior Iliacus (RPSI), were used to calculate vertical displacements of the pelvis during turning. To do that, the difference between the vertical positions of each marker measured at the beginning and end of the turning was obtained and normalized by the duration of the turning and by the length of the subject's leg. The duration of the turning part of the task was determined from the moment of the toe-off of the second step to the toe-off of the fourth step (Figure 2). Three trials were recorded and analyzed for each experimental condition.

Symmetry indexes (SI) were calculated using the normalized values of the position of the LASI and RASI (frontal symmetry) and LPSI and RPSI (dorsal symmetry) and the following equation [20]:

(1) $$\begin{array}{c} SI=\frac{V_{contralateral}-V_{target}}{\left(1/2\right)\left(V_{contralateral}+V_{target}\right)}\times 100, \end{array}$$

where *V*_target_ is the corresponding variable for the limb provided with the insole (right leg) and *V*_contralateral_ is a variable recorded for the contralateral limb (not provided with the insole, i.e., left leg). A SI = 0 represents perfect symmetry.

**Figure 3:**
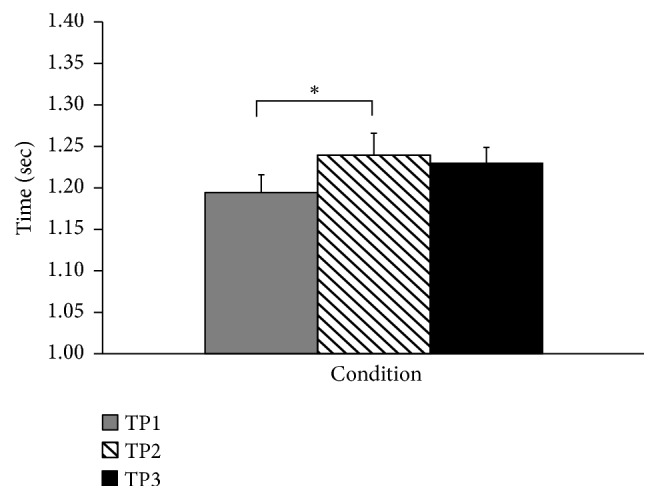
Turning time. Significant difference (*p* < 0.05) is shown with the asterisk. TP1 is turning with no insole. TP2 turning immediately after being provided with the insole and TP3 turning after 6 min of walking with the insole.

**Figure 4:**
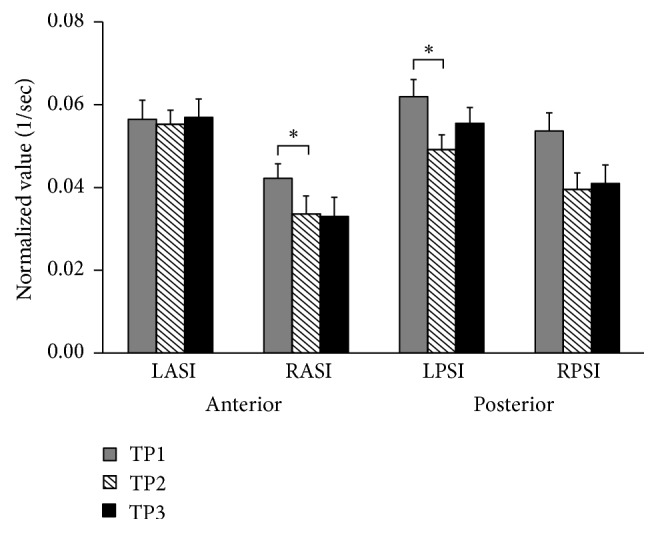
Normalized displacements of the Left Anterior Superior Iliacus (LASI), Right Anterior Superior Iliacus (RASI), Left Posterior Superior Iliacus (LPSI), and Right Posterior Superior Iliacus (RPSI) pelvic markers are shown. Each column represents the difference between the vertical positions of the same marker measured at the beginning and end of the turning normalized by the duration of turn and leg length. Significant difference (*p* < 0.05) is shown with asterisks.

**Figure 5:**
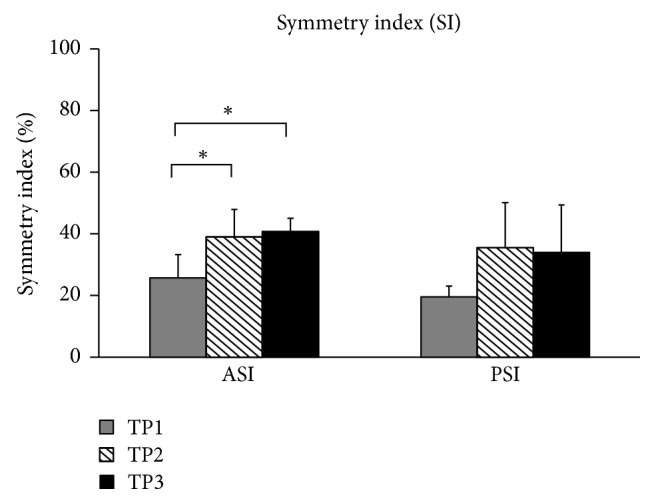
Symmetry indexes calculated for the frontal (anterior) and dorsal (posterior) parts of the body. Significant difference (*p* < 0.05) is shown with asterisks.

**Figure 6:**
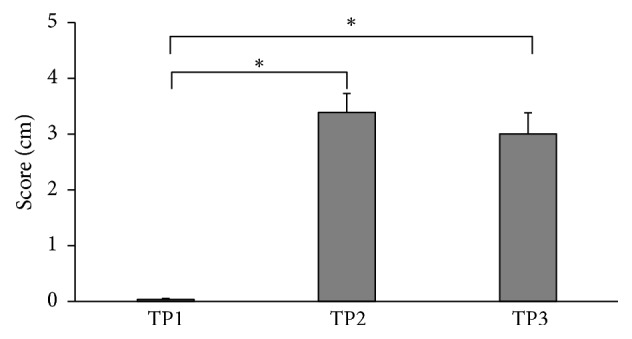
Visual Analog Scale (VAS) scores obtained from all the subjects at three time points. Significant difference (*p* < 0.05) is shown with asterisks.

One-way repeated-measures ANOVAs were used to compare each outcome variable (the duration of turning, normalized displacement of markers, SI, and VAS) between the three experimental conditions (TP1, TP2, and TP3). Statistical analysis was performed in SPSS Version 22 (IBM, USA). Statistical difference was set at *p* < 0.05. Post hoc tests were performed where there was a main effect of time. Means and standard errors are presented in the Results and Figures 3–6.

## 3. Results

### 3.1. Duration of Turning

The duration of the turning in no insole condition (TP1) was 1.19 ± 0.02 sec; when wearing a textured insole (TP2), the duration of turning increased to 1.24 ± 0.03 sec. The difference was statistically significant (*p* = 0.048). The duration of turning after walking with the insole for six minutes (TP3) was 1.23 ± 0.02 sec. The difference in timing between TP1 and TP3 and between TP2 and TP3 was not significant (Figure 3).

### 3.2. Pelvis Displacement

The displacements of pelvic markers in the vertical plane are shown in Figure 4. The vertical position of all four markers was the highest in the TP1 condition as compared to the TP2 and TP3. The largest difference between the conditions could be seen in the displacements of the LPSI, RPSI, and RASI markers. The difference between the experimental conditions of turning with no insole (TP1) and turning with the insole (TP2) was statistically significant for the RASI and LPSI markers (*p* < 0.05). After walking with the insole for six minutes (TP3), the vertical positions of LPSI and RPSI markers during the turns were slightly higher than the positions of markers measured while in the TP2 condition (immediate effect of the insole). However, the difference was not statistically significant.

### 3.3. Symmetry of Turning

Symmetry indexes (SI) for the frontal part of the pelvis were 25.69 ± 7.54 when turning with no insole. The body asymmetry increased when walking and turning with the textured insole (TP2) reaching 38.99 ± 8.90. The difference was statistically significant (*p* < 0.05). When turning after walking for six minutes (TP3), the body asymmetry increased to 40.75 ± 4.27. This level of asymmetry was significantly different from the body asymmetry while turning in no insole condition (*p* < 0.05) (Figure 5).

Symmetry index (SI) for the dorsal part of pelvis was 19.51 ± 3.52 when turning with no insole. The body asymmetry increased in the TP2 reaching 35.50 ± 14.61. The difference, however, was not statistically significant. When turning after walking for six minutes, the body asymmetry decreased to 33.97 ± 15.4 but it still was higher than in TP1 condition.

### 3.4. Perceived Level of Discomfort

Changes in the level of the perceived discomfort are presented in Figure 6. VAS score while walking and turning with no insole was 0.04 ± 0.02 cm. When the subjects performed the task using the textured insole, their perceived level of discomfort increased to 3.39 ± 0.34 cm and to 3.00 ± 0.37 cm when measured after walking with the insole for six minutes. The difference was statistically significant between the discomfort level in no insole (TP1) condition and that immediately after the start of using the insole (TP2) (*p* < 0.001). The difference between conditions with no insole (TP1) and after walking with the insole for six minutes (TP3) was statistically significant (*p* < 0.001). The difference between TP2 and TP3 conditions was not statistically significant.

## 4. Discussion

Discomfort induced on one side of the body could alter movement performance [10]. Furthermore, it was shown that healthy individuals demonstrate asymmetry of static and dynamic posture and gait immediately after they are provided with the insole that induces discomfort [12]. The current study aimed to investigate whether a single textured insole could alter the performance of the turning task. The outcome demonstrated that walking and turning with the textured insole placed in one shoe was associated with increased asymmetry of the position of the pelvis. Thus, the hypothesis that discomfort created by the textured insole would cause asymmetry of turning in healthy individuals was supported.

It was reported in the literature that individuals with stroke perform the turning task slower, spending more time to accomplish the turning, than the healthy controls [21]. The use of a textured insole in the current study was associated with slower performance of the turning task in healthy subjects which is confirmed by the increased duration of the turning in condition with the insole. This outcome is in contrast with the outcome of a previous study demonstrating that gait velocity was not affected by the insole [12]. A possible explanation of this dissimilarity could be that there are differences between the tasks: turning to the right with the insole placed in the right sandal created the level of discomfort higher than the subjects experienced while walking straight [12]. Indeed, the VAS scores reported by the subjects in the current study were above 3 cm while in the previous study the scores were below 2 cm [12]. It looks like, due to the increased level of discomfort, the subjects in the current study performed the turning task slower and more cautiously, resembling performance of the task by individuals with stroke [2].

There were some differences in the normalized displacements of pelvic markers in the vertical plane between the experimental conditions. For example, smaller displacements were observed when turning with the insole (TP2) as compared to turning with no insole (TP1); this could be due to the effect of discomfort induced by an insole, preventing a subject from shifting body weight to the right side while turning. On the other hand, the observed slight increase in the displacement of pelvic markers, especially LPSI, after walking with the insole for six minutes (TP3) could be because of the habituation to the induced discomfort. This possible explanation is in line with prior literature describing adaptation of the somatosensory receptors on the plantar surface of the foot in response to the constant tactile stimulus provided by the textured insole [12].

Placing a textured insole in the shoe of healthy individuals is associated with gait asymmetry [10, 12] which is commonly observed in individuals with stroke-related hemiparesis [22]. The subjects in the current study, when provided with a textured insole, demonstrated increased level of asymmetry of turning resembling a commonly seen asymmetry of movement performance in individuals with unilateral stroke [22]. Consequently, the outcomes of previous studies reporting a single insole-related minimization of the asymmetry of stance and gait in individuals with unilateral impairment [9, 10, 13, 23, 24] together with the results of the current study allow assuming that improvement in turning of individuals with stroke could be seen when they are provided with the textured insole in the shoe on the healthy side.

The study participants reported increased level of discomfort while turning with the textured insole in the right shoe. The reported level of perceived discomfort was significantly higher when they were provided with the textured insole compared to the no insole condition. Thus, the results of the VAS tests suggest that the use of a textured insole was indeed associated with an increased level of perceived discomfort. At the same time, the subjects did not report any problems related to using the textured insole, which suggests that they tolerated the induced discomfort well. As such, this study outcome advocates for a possibility that individuals with unilateral impairment would be able to use the textured insole as well. Prior literature supports this assumption [10, 13].

The study has several limitations. First, while the outcomes show that using a single textured insole induces asymmetry of turning in healthy individuals, the findings cannot be extrapolated to clinical populations. Second, the sample size was small and we did not compare the performance of healthy individuals with individuals with neurological disorders. Third, we studied only 90-degree turning to the right side and put the textured insole in the shoe on the dominant side. Future study should be conducted with a larger sample size and involve individuals with unilateral impairment performing turning in different directions.

## 5. Conclusion

The results of the study indicate that using a single textured insole induces significant changes in the symmetry of turning in healthy individuals. This outcome provides a basis for future investigations of the efficacy of the textured insole in minimizing asymmetry of turning in individuals with unilateral impairments such as stroke.
